# A statistical model of COVID-19 testing in populations: effects of sampling bias and testing errors

**DOI:** 10.1098/rsta.2021.0121

**Published:** 2022-01-10

**Authors:** Lucas Böttcher, Maria R. D'Orsogna, Tom Chou

**Affiliations:** ^1^ Department of Computational Medicine, University of California, Los Angeles, 90095-1766 Los Angeles, CA, USA; ^2^ Department of Mathematics, University of California, Los Angeles, 90095-1766 Los Angeles, CA, USA; ^3^ Computational Social Science, Frankfurt School of Finance and Management, 60322 Frankfurt am Main, Germany; ^4^ Department of Mathematics, California State University at Northridge, Los Angeles, 91330-8313 CA, USA

**Keywords:** COVID-19, testing, combinatorics

## Abstract

We develop a statistical model for the testing of disease prevalence in a population. The model assumes a binary test result, positive or negative, but allows for biases in sample selection and both type I (false positive) and type II (false negative) testing errors. Our model also incorporates multiple test types and is able to distinguish between retesting and exclusion after testing. Our quantitative framework allows us to directly interpret testing results as a function of errors and biases. By applying our testing model to COVID-19 testing data and actual case data from specific jurisdictions, we are able to estimate and provide uncertainty quantification of indices that are crucial in a pandemic, such as disease prevalence and fatality ratios.

This article is part of the theme issue ‘Data science approach to infectious disease surveillance’.

## 1. Introduction

Real-time estimation of the level of infection in a population is important for assessing the severity of an epidemic as well as for guiding mitigation strategies. Several previous studies have addressed the issue of correcting for errors and testing biases. However, inferring disease prevalence via patient testing is challenging due to testing inaccuracies, testing biases and heterogeneous and dynamically evolving populations and severity of the disease.

There are two major classes of tests that are used to detect previous and current SARS-CoV-2 infections [1]. Serological, or antibody, tests measure the concentration of antibodies in infected and recovered individuals. Since antibodies are generated as a part of the adaptive immune system response, it takes time for detectable antibody concentrations to develop. Serological tests should thus not be used as the only method to detect acute SARS-CoV-2 infections. An alternative testing method is provided by viral-load or antigen tests, such as reverse transcription polymerase chain reaction (RT-PCR), enzyme-linked immunosorbent assay (ELISA) and rapid antigen tests, which are able to identify ongoing SARS-CoV-2 infections by directly detecting SARS-CoV-2 nucleic acid or antigen.

Test results are mainly reported as binary values (0 or 1, negative or positive) and often do not include further information such as the cycle threshold (Ct) for RT-PCR tests. The cycle threshold Ct defines the minimum number of PCR cycles at which amplified viral RNA becomes detectable. Large values of Ct indicate low viral loads in the specimen. An increase in Ct by a factor of about 3.3 corresponds to a viral load that is about one order of magnitude lower [2]. Cycle threshold cutoffs are not standardized across jurisdictions and range from values between 37 and 40, making it difficult to compare RT-PCR test results [3]. Lower Ct cutoffs in the range of 30–35 may be more reasonable to avoid classifying individuals with insignificant viral loads as positive [3].

Further uncertainty in COVID-19 test results arises from different type I errors (false positives) and type II errors (false negatives) that are associated with different assays. Note that inherent to any test, the threshold (such as Ct mentioned above) may be tunable. Therefore, besides intrinsic physical limitations, binary classification of ‘continuous-valued’ readouts (e.g. viral load) may also lead to an overall error of either type [4]. In this work, we will assume that there is a standardized threshold and the test readout is binary; if any virus is detected, the test subject is positive. We will not explicitly model the underlying statistics of the errors but assume that the test readouts are binary but can be erroneous at specified rates. Some uninfected individuals will be wrongly classified as infected with rate FPR and some infected individuals will be wrongly classified as uninfected with rate FNR. For serological COVID-19 tests, the estimated proportions of false positives and false negatives are relatively low, with FPR≈0.02−0.07 and FNR≈0.02−0.16 [5–8]. The FNRs of RT-PCR tests depend strongly on the actual assay method [9,10] and may be significantly larger than those of serological tests. Typical values of FNR for RT-PCR tests lie between 0.1 and 0.3 [11,12] but might be as high as FNR≈0.68 if throat swabs are used [7,12]. False-negative rates may also vary significantly depending on the time delay between initial infection and testing [8]. According to a systematic review [13] that was conducted worldwide, the initial value of FNR is about 0.54, underlying the importance of retesting. Similar to serological tests, reported false-positive rates of RT-PCR tests are about FPR=0.05 [7].

Estimates of disease prevalence and other surveillance metrics [14,15] need to account for FPRs and FNRs, in particular if reported positive-testing rates [16] are in the few percent range and potentially dominated by type I errors. In addition to type I/II testing errors, another confounding effect is biased testing [17], that is preferential testing of individuals that are expected to carry a high viral load (e.g., symptomatic and hospitalized individuals). Biasing testing towards certain demographic and risk groups leads to additional errors in disease prevalence estimates that need to be corrected for.

In §2, we discuss related studies that developed statistical methods to correct for erroneous and biased testing. To account for type I/II errors, bias, retesting and exclusion after testing, we develop a corresponding framework for disease testing in §3. We apply our testing model to COVID-19 testing and case data in §4 and estimate testing bias by comparing random-sampling testing data [18] with officially reported, biased COVID-19 case data in §5. We conclude our study in §6.

## 2. Related work

Several previous studies have addressed the issue of correcting for errors and testing biases. In the random-sampling study [18], specificity and sensitivity corrected ELISA results are reported without specifying the actual statistical correction method. In another work [19], corrected case numbers for different European countries are derived based on the assumption that the infection fatality ratio (IFR) is independent of the geographical location. If the IFR were known exactly, this method could be used to estimate the sampling bias by comparing the reported number of cases with the corresponding reported number of deaths divided by IFR. However, the framework in [19] does not account for false negatives and false positives. In addition, there are geographical variations of the IFR that may be attributed to significant differences in incidence rates, population density, preparedness of public health systems and age structure [14,20,32]. Therefore, the assumption of a time and location-independent IFR may yield inaccurate results.

In [21], a semi-Bayesian probabilistic bias analysis is used to estimate the cumulative number of SARS-CoV-2 infections in the United States. The employed corrections for erroneous testing are similar to the results that we derive in §4. Corrections for incomplete testing are based on distributions associated with random sampling studies similar to [18], which we use in §5.

One major difference between [21] and our work is that we derive the distributions with and without retesting, and explicitly account for test-type-dependent specificities and sensitivities.

## 3. Statistical testing model

Here, and in the following subsections, we develop a general statistical model for estimating the number of infected individuals in a jurisdiction by testing a sample population. The relevant variables and parameters to be used in our derivations are listed and defined in table 1. Suppose we randomly administer Q tests within a given short time period (e.g. within 1 day or 1 week) to a total effective population of N previously untested individuals. This population comprises S susceptible, I infected and R removed (i.e. recovered or deceased) individuals, which are unknown. S, I and R can dynamically change from one testing period to another due to transmission and recovery dynamics, as well as removal from the untested pool by virtue of being tested. The total population N=S+I+R can also change through intrinsic population dynamics (birth, death and immigration), but can assumed to be constant over the typical time scale of an epidemic that does not cause mass death.

**Table 1.** Overview of variables used in testing model. An overview of the main variables and parameters that will be used in developing our testing model. The sets [0,N] and [0,Q] contain all integers from 0 up to Q and N, respectively. The set $f,f_{b},{\tilde{f}}_{b}:[0,1]$ denotes all rational numbers between 0 and 1. For FNR,FPR, [0,1] represents all real numbers between 0 and 1. We assume that Q, $Q^{+}$, ${\tilde{Q}}^{+}$, $f_{b}:=Q^{+}/Q$ and ${\tilde{f}}_{b}:={\tilde{Q}}^{+}/Q$ are determined by testing a population of known size N.

symbol	definition
$N\in Z^{+}$	population in jurisdiction
Q:[0,N]	number of tests administered
$Q^{+}:[0,Q]$	recorded positives under error-free testing
${\tilde{Q}}^{+}:[0,Q]$	recorded positives under error-prone testing
f:[0,1]	true proportion of infected individuals
$f_{b}:=Q^{+}/Q:[0,1]$	fraction of positives under biased, error-free testing
${\tilde{f}}_{b}:={\tilde{Q}}^{+}/Q:[0,1]$	fraction of positives under biased, error-prone testing
b∈R	testing bias parameter
$\hat{b},\hat{f}:[0,1]$	estimates of bias and underlying infection fraction
FPR:[0,1]	false-positive rate
FNR:[0,1]	false-negative rate

We start the derivation of our statistical model by first fixing S, I and R, assuming both perfect error-free testing, considering a ‘testing with replacement’ scenario, in which tested individuals can be retested within the same time window. Under these conditions, the probability that q tests are returned positive and $Q^{-}=Q-q$ tests are returned negative is

3.1 Ptrue(Q+=q|Q,S,I,R)=(Qq)fq(1−f)Q−q,

where the parameter

3.2 $$f\equiv \frac{\left(I+R\right)}{N}\;\text{or}\;\frac{I}{N},$$

is the probability of identifying currently and previously infected individuals with tests such as serological (antibody) tests, or of detecting current infections with viral load tests, respectively. Note that testing with replacement renders $P_{\text{true}}$ dependent only on Q and f, and not explicitly on I,S,R or N. The binomial expression (3.1) is accurate when the number of tests are much smaller than the population (I+R) or I.

Equation (3.1) describes perfect error-free and random testing. However, if there is some prior suspicion of being infected, the administration of testing may be biased. For example, certain jurisdictions focus testing primarily on hospitalized patients and people with significant symptoms [17], thus biasing the tests to those that are infected. We quantify such testing biases through a biased-testing function $K(Q^{+})\in R_{\geq 0}$, leading to the following modification of equation (3.1):

3.3 Ptrue(Q+=q|Q,f)=(Qq)fq(1−f)Q−qK(q)∑k=0Q(Qk)fk(1−f)Q−kK(k).

We define the biased-testing function K(q) as a weight over the number of returned q positive and Q−q negative tests. A convenient choice is $K(q)={\text{e}}^{qU_{I}}\;{\text{e}}^{(Q-q)U_{S}}$, which weights each of the q positive tests from actually infected patients, by the factor ${\text{e}}^{U_{I}}$, and each of the Q−q negative tests, from non-infected, susceptible persons, by the factor ${\text{e}}^{U_{S}}$. The bias is implicitly defined via the quantity $b=U_{I}-U_{S}$, so that if b≠0 the two test results carry different weights, with b>0 favouring infected patients over non-infected persons, and vice versa for b<0. The quantities ${\text{e}}^{U_{I}}$, ${\text{e}}^{U_{S}}$ can also be interpreted as costs to administer tests to infected and susceptible persons, respectively. The inclusion of K(q) in $P_{\text{true}}(Q^{+}=q|f,Q)$ yields

3.4 Ptrue(Q+=q|Q,f,b)=(Qq)fq(1−f)Q−q eqb∑k=0Q(Qk)fk(1−f)Q−k ekb=(Qq)(f eb)q(1−f)Q−q[1+(eb−1)f]Q,

A Gaussian approximation to equation (3.4) can be found using the de Moivre–Laplace theorem [22], which leads to

3.5 $$P_{\text{true}}(Q^{+}=q|Q,f,b)\approx \frac{1}{\sigma \sqrt{2\pi }}\exp\left[-\frac{{\left(q-\mu (f,b)\right)}^{2}}{2\sigma^{2}(f,b)}\right],$$

where

3.6 $$\mu (f,b)\equiv \frac{Qf\;{\text{e}}^{b}}{1+({\text{e}}^{b}-1)f}\;\text{and}\;\sigma^{2}(f,b)\equiv \mu (f,b)\left(1-\frac{\mu (f,b)}{Q}\right).$$

In addition to describing the probability distribution in equation (3.5) as a function of the number of positive tests $Q^{+}$, we can also express it in terms of the observed positive (and potentially sample-biased) testing fraction $f_{\text{b}}:=Q^{+}/Q$:

3.7 $$P_{\text{true}}(f_{\text{b}}=x|Q,f,b)\approx \frac{1}{\bar{\sigma }(f,b)\sqrt{2\pi }}\exp\left[-\frac{{\left(x-\bar{\mu }(f,b)\right)}^{2}}{2{\bar{\sigma }}^{2}(f,b)}\right],$$

where here

3.8 $$\bar{\mu }(f,b)\equiv \mu /Q=\frac{f\;{\text{e}}^{b}}{1+({\text{e}}^{b}-1)f}\;\text{and}\;{\bar{\sigma }}^{2}(f,b)\equiv \bar{\mu }(f,b)(1-\bar{\mu }(f,b))/Q.$$

A standard rule for validity of the de Moivre–Laplace theorem is to assert that the product of sample population, success probability, and its counter probability is larger than 9 [23]; in this case, equations (3.5) and (3.7) will hold if μ(f,b)(1−μ(f,b)/Q)>9 or equivalently if $\bar{\mu }(1-\bar{\mu })Q>9$.

The expected value of $f_{\text{b}}$, $\bar{\mu }(f,b)$, can be understood as a product of the true underlying infected fraction f and a bias function B(f,b) that depends on f and the bias parameter b, i.e. $\bar{\mu }(f,b)=fB(f,b)$, where B(f,b) is given by

3.9 $$B(f,b)=\frac{{\text{e}}^{b}}{1+({\text{e}}^{b}-1)f}.$$

Note that equation (3.9) implies that when b>0, the currently and/or previously infected population is favoured to be tested, while for b<0, the non-infected and/or susceptible population is favoured. The limits of b→±∞ indicate testing that is completely biased such that only infected and susceptible individuals are tested, respectively. Realistic values of our bias parameter b are positive and ∼O(1).

**Figure 1.**
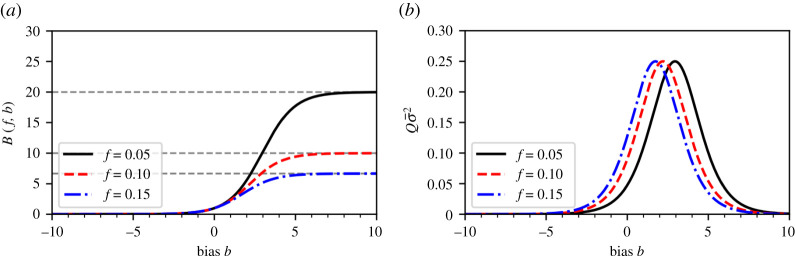
Illustration of a bias function B(f,b). (*a*) The bias function B(f,b) (equation (3.9)) for three different fractions f of currently (and previously) infected individuals. Grey dashed lines indicate the asymptotic value B(f,∞)=1/f. A value b>0 indicates a testing bias towards currently and/or previously infected individuals while susceptible and/or non-infectious individuals are preferentially tested for b<0. Unbiased testing corresponds to b=0 and B(f,0)=1/f. (*b*) The variance ${\bar{\sigma }}^{2}$ exhibits a maximum value of 1/(4Q) at a typical value of bias $b^{*}=\ln[(1-f)/f]$. (Online version in colour.)

Figure 1*a* shows the bias function (3.9) as a function of b for different infection fractions f. For B(f,b)>1, the biased-testing fraction fB is larger than the unbiased-testing fraction f. The opposite holds for B(f,b)<1. If only susceptible individuals are tested (i.e. b→−∞), the bias function B(f,b) and the expected observed positive testing fraction $\bar{\mu }(f,b)=fB(f,b)$ approach zero. For a complete bias towards infected individuals (i.e. b→∞), the bias function approaches $f^{-1}$ and $\bar{\mu }(f,b)\to 1$.

The variance ${\bar{\sigma }}^{2}$ of the Gaussian approximation (3.7) is plotted as a function of b in figure 1*b* and exhibits a maximum value of 1/(4Q) at $b^{*}=\ln[(1-f)/f]$.

The probabilities $P_{\text{true}}$ derived in equations (3.1) and (3.3) correspond to ‘testing with replacement’. The opposite limit is ‘testing without replacement’; once an individual is tested they are labelled as such and removed from the pool of test targets, at least within the specified testing period. This concept of sampling with and without replacement commonly arises in the measurement of diversity in ecological settings [24]. Without replacement, and still under conditions of perfect random testing, two slightly different forms for $P_{\text{true}}$ arise for the different type of tests (e.g. antibody versus PCR/viral load). For antibody tests that perfectly identify recovered (or deceased) individuals as being previously infected, equation (3.1) is replaced by

3.10 Ptrue(Q+=q|S,I,R,Q)=(I+Rq)(SQ−q)(NQ)=(Qq)∏n=0Q−1(1N−n)∏i=0q−1(I+R−i)∏j=0Q−q−1(S−j),

where the binomial coefficients for q=0,Q are zero. On the other hand, if the perfect test only identifies individuals that currently carry a viral load, the susceptible and recovered (or deceased) individuals both test negative, and $P_{\text{true}}$ is described by

3.11 Ptrue(Q+=q|S,I,R,Q)=(Iq)(S+RQ−q)(NQ)=(Qq)∏n=0Q−1(1N−n)∏i=0q−1(I−i)∏j=0Q−q−1(S+R−j).

The expressions for $P_{\text{true}}$ when tested subjects are not replaced, unlike in the case of testing with replacement, depend explicitly on S,I,R and N.

To incorporate testing bias into the probabilities $P_{\text{true}}$ for testing without replacement, we first consider equation (3.11) where (Iq)(S+RQ−q) can be interpreted as the number of ways of distributing q positive tests among I infected individuals, and Q−q negative tests among S+R uninfected individuals. As in the biased-testing formulation of equation (3.3), we interpret the bias as a factor K(q) that assigns more weight to tests in the I or S+R pools

3.12 Ptrue(Q+=q|Q,S,I,R,b)≡(Iq)(S+RQ−q)K(q)∑k=0Q(Ik)(N−IQ−k)K(k).

To obtain the ‘testing without replacement’ equivalent of equation (3.4), we again set $K(q)={\text{e}}^{qU_{I}}\;{\text{e}}^{(Q-q)U_{S}}={\text{e}}^{bq}\;{\text{e}}^{QU_{S}}$. By using the Chu–Vandermonde identity

3.13 ∑k=0Q(Ik)(N−IQ−k)=(NQ),

we verify that when b=0 equation (3.12) reduces to equation (3.11).

The above choice for K(q) also allows us to explicitly evaluate the denominator in equation (3.12)

3.14 ∑k=0Q(Ik)(N−IQ−k)ekb=(N−IQ) 2F1(−I,−Q;N−I−Q+1;eb),

where ${}_{2}F_{1}$ denotes the (ordinary) hypergeometric function. Thus, the distribution of positive tests $Q^{+}$ under biased testing without replacement for viral load-type tests can be expressed as

3.15 Ptrue(Q+=q|Q,S,I,R,b)≡eqb(Qq)∏k=0q−1(I−kS+R−Q−−k) 2F1(−I,−Q;N−I−Q+1;eb).

The distribution of positives under biased testing without replacement for antibody-type tests (using equation (3.10) as a starting point) is

3.16 Ptrue(Q+=q|Q,S,I,R,b)≡eqb(Qq)∏k=0q−1(I+R−kS−Q−−k) 2F1(−I−R,−Q;N−I−R−Q+1;eb),

where $Q^{-}\equiv Q-q$. The above two expressions are equivalent except for the merging of the R pool with uninfected susceptibles S in one case, or with current infecteds I in the other. Because the testing decreases with the number of tests administered, equations (3.15) and (3.16) cannot be reduced to functions of a simple positive-test fraction $f_{\text{b}}$ or to simple Gaussian forms.

### (a) Testing errors

The probability distributions $P_{\text{true}}$ that we derived in equation (3.3) and in equations (3.10)–(3.11) assume that testing is error-free, i.e. that the false-negative rate FNR=1−TPR=0 and false-positive rate FPR=1−TNR=0, or equivalently that the true positive rate TPR=1 and the true negative rate TNR=1. To incorporate erroneous testing, we now construct the probability distribution of error-generated deviation $P_{\text{err}}({\tilde{Q}}^{+}=q|Q^{+}=q^{\prime })$ over the number of ‘apparent’ positives q from tests that carry nonzero FPRs and FNRs, given that $q^{\prime }$ positives would be recorded if the tests were perfect. If q apparent positive tests are tallied, p of them might have been true positives drawn from the perfect-test positives $q^{\prime }$ in (q′p) ways, while the remaining k=q−p apparent positives might have been erroneously counted as positives drawn from the $Q^{-}\equiv Q-q^{\prime }$ true negatives. The remaining $q^{\prime }-p$ true positive tests might have been erroneously tallied as false negatives, while the remaining $Q^{-}-k$ negative tests might have been correctly tallied as true negatives. Assuming non-zero FPR and FNR, we find that the probability distribution of finding 0≤q≤Q apparent positive tests is

3.17 Perr(Q~+=q|Q+=q′,Q,FPR,FNR)=∑p=0q(q′p)(TPR)p(FNR)q′−p(Q−q′q−p)(FPR)q−p(TNR)Q−q′−(q−p),

where we invoked the identities TPR+FNR=1 and FPR+TNR=1. The total error-prone distribution $P_{\text{TOT}}({\tilde{Q}}^{+}=q|Q,S,I,R,b)$ of recording q positives after having administered Q tests under bias and/or testing errors is given by convolving the probability $P_{\text{err}}$ of finding q apparent tests given $q^{\prime }$ true positive tests with the probability $P_{\text{true}}$ of finding $q^{\prime }$ positive tests under perfect testing (equation (3.4) or equation (3.15)):

3.18 $$\begin{array}{rl} & P_{\text{TOT}}({\tilde{Q}}^{+}=q|Q,S,I,R,b,\text{FPR},\text{FNR})\\ & \;=\sum_{q^{\prime }=0}^{Q}P_{\text{err}}({\tilde{Q}}^{+}=q|Q^{+}=q^{\prime },Q,\text{FPR},\text{FNR})P_{\text{true}}(Q^{+}=q^{\prime }|Q,S,I,R,b). \end{array}$$

This convolution can be further simplified by taking the Gaussian limit of the binomial distributions that appear in $P_{\text{err}}$ and in $P_{\text{true}}$, once more invoking the de Moivre–Laplace theorem [22]. To be concrete, we use $P_{\text{true}}$ as written in equation (3.3) under the testing with replacement scenario and use the approximation (3.5). The same Gaussian approximation can be used for both binomial terms in $P_{\text{err}}$ from equation (3.17), under the conditions illustrated above, which yield $\text{TPR}(1-\text{TPR})Q^{+}>9$, $\text{FPR}(1-\text{FPR})(Q-Q^{+})>9$. These will complement the condition $\bar{\mu }(1-\bar{\mu })Q>9$ that arises from using equation (3.5) in lieu of equation (3.17). Once the summand in equation (3.17) has been expressed as a product of two Gaussians, we approximate the summation over p∈(0,q) by an integral over p∈(−∞,∞). Provided q is sufficiently large, we find

3.19 $$P_{\text{err}}({\tilde{Q}}^{+}=q|Q,Q^{+}=q^{\prime },\text{FPR},\text{FNR})\approx \frac{\exp\left[-\frac{{\left(q-q^{\prime }\text{TPR}-(Q\;-\;q^{\prime })\text{FPR}\right)}^{2}}{2q^{\prime }\text{FNR}\;\text{TPR}+2(Q\;-\;q^{\prime })\text{FPR}\;\text{TNR}}\right]}{\sqrt{2\pi }\sqrt{q^{\prime }\text{FNR}\;\text{TPR}+(Q\;-\;q^{\prime })\text{FPR}\;\text{TNR}}}.$$

We now convolve equation (3.5) with equation (3.19) as prescribed by equation (3.18) to find

3.20 $$P_{\text{TOT}}({\tilde{Q}}^{+}=q|Q,f,b,\text{FPR},\text{FNR})\approx \frac{1}{\sigma_{\text{T}}\sqrt{2\pi }}\exp\left[-\frac{{\left(q-\mu_{\text{T}}\right)}^{2}}{2\sigma_{\text{T}}^{2}}\right],$$

where

3.21 $$\begin{array}{rl} \mu_{\text{T}}(f,b,\text{FPR},\text{FNR}) & \equiv Q[\bar{\mu }(f,b)(1-\text{FNR})+(1-\bar{\mu }(f,b))\text{FPR}],\\ \sigma_{\text{T}}^{2}(f,b,\text{FPR},\text{FNR}) & \equiv Q(1-\bar{\mu }(f,b))\text{FPR}(1-\text{FPR})+Q\bar{\mu }(f,b)\text{FNR}(1-\text{FNR})\\ & \;+Q\bar{\mu }(f,b)(1-\bar{\mu }(f,b)){\left(1-\text{FNR}-\text{FPR}\right)}^{2}, \end{array}\}$$

with $\bar{\mu }(f,b)$ the mean value of the fraction of observed positives under biased, but perfect testing (see equation (3.8)). To derive equation (3.20), we replaced the summation over $q^{\prime }\in (0,Q)$ by an integral over $q^{\prime }\in (-\infty ,\infty )$. This approximation is valid if the value of the two Gaussians that approximate $P_{\text{err}}$ and $P_{\text{true}}$ in equation (3.18) are negligible when evaluated at $q^{\prime }\leq 0$ and $q^{\prime }\geq Q$ for all values of q. We have verified that this is true provided that 0<2/(Q+2)≪FPR, FNR, $\bar{\mu }\ll Q/(Q+2)<1$. In the limit of large numbers of tests Q, this condition will almost always be satisfied. Finally, to perform the integral that now appears in equation (3.18), we fixed the value of $q^{\prime }=\bar{\mu }$ in the $q^{\prime }$-dependent variance in equation (3.19). We do this to obtain an analytic expression for the integral of $P_{\text{err}}P_{\text{true}}$ over $q^{\prime }$, and select the value $q^{\prime }=\bar{\mu }$ for the variance of $P_{\text{err}}$ as the one that carries the most weight from $P_{\text{true}}$.

Equation (3.20) reveals that the mean number of apparent positive tests $\mu_{\text{T}}$ is given by the sum of the expected value of true positive tests (i.e. $Q\bar{\mu }(f,b)(1-\mathrm{FNR})=Q\bar{\mu }(f,b)\mathrm{TPR}$) and the expected value of false positive tests (i.e. $Q(1-\bar{\mu }(f,b))\mathrm{FPR}$). Based on the derived expressions for $\mu_{\text{T}}$ and $\sigma_{\text{T}}^{2}$, we define the random variable as the fraction of observed positive tests ${\tilde{f}}_{\text{b}}\equiv {\tilde{Q}}^{+}/Q$ under biased and error-prone testing and obtain

3.22 $$P_{\text{TOT}}({\tilde{f}}_{\text{b}}=x|Q,f,b,\text{FPR},\text{FNR})\approx \frac{{\text{e}}^{-{\left(x-{\bar{\mu }}_{\text{T}}\right)}^{2}/(2{\bar{\sigma }}_{\text{T}}^{2})}}{{\bar{\sigma }}_{\text{T}}\sqrt{2\pi }},$$

where

3.23 $$\begin{array}{rl} & {\bar{\mu }}_{\text{T}}(f,b,\text{FPR},\text{FNR})\equiv [\bar{\mu }(f,b)(1-\text{FNR})+(1-\bar{\mu }(f,b))\text{FPR}]\\ \;\text{and}\; & Q{\bar{\sigma }}_{\text{T}}^{2}(f,b,\text{FPR},\text{FNR})\equiv (1-\bar{\mu }(f,b))\text{FPR}(1-\text{FPR})+\bar{\mu }(f,b)\text{FNR}(1-\text{FNR})\\ & \;\;\;\;\;+\bar{\mu }(f,b)(1-\bar{\mu }(f,b)){\left(1-\text{FNR}-\text{FPR}\right)}^{2}. \end{array}\}$$

**Figure 2.**
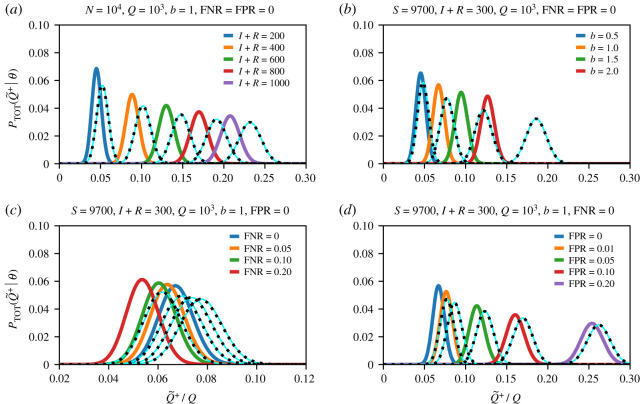
Distribution of apparently positive tests. Plots of $P_{\text{TOT}}({\tilde{Q}}^{+}=q|Q,I,S,R,b)$ with $N=S+I+R=10^{4}$, $Q=10^{3}$, and different (*a*) values of I+R, (*b*) testing biases b, (*c*) FNRs, and (*d*) FPRs. The Gaussian approximation (solid light blue lines) of equation (3.22) provides an accurate approximation of $P_{\text{TOT}}$. Dashed black lines correspond to distributions with replacement and the remaining thicker solid coloured lines correspond to those without replacement. (Online version in colour.)

We have numerically verified that the Gaussian approximation (3.23) is quite accurate provided that (i) the number of positive and apparent positive tests, $Q^{+}=q^{\prime }$ and ${\tilde{Q}}^{+}=q$, are sufficiently large, and (ii) the quantities $\bar{\mu }$, FNR and FPR are not too close to 0 or 1. This is shown in figure 2 and is in accordance with the conditions implied by using the de Moivre–Laplace theorem and by exchanging the sum with the integration when evaluating equation (3.18), as described above. Figure 2 shows the distribution of apparently infected individuals $P_{\text{TOT}}$ for different numbers of infected/recovered individuals (figure 2*a*), testing biases (figure 2*b*), and testing sensitivities (i.e. true positive rates, TPRs) and specificities (i.e. true negative rates, TNRs) (figure 2*c*–*d*). Solid light blue lines (colour available in online version) represent the Gaussian approximation (3.22) and dashed black lines and the remaining thicker coloured lines are calculated by directly evaluating equation (3.18) with replacement (equation (3.3)) and without replacement (equation (3.16)), respectively. The FNRs that we consider in figure 2*c* are chosen in accordance with reported sensitivities of serological and RT-PCR tests for SARS-CoV-2 [9–12]. We observe that an increase in the FNR slightly shifts the distribution $P_{\text{TOT}}$ towards smaller values of apparently infected individuals, which is consistent with the FNR dependence of the mean ${\bar{\mu }}_{\text{T}}$ (equation (3.23)). For serological and RT-PCR tests, the FPR=1−TNR is about 5%. A smaller specificity would lead to larger FPRs and a shift of $P_{\text{TOT}}$ towards larger values of ${\tilde{Q}}^{+}$ and ${\tilde{f}}_{\text{b}}$ (figure 2*d*). Our results show that $P_{\text{TOT}}$ is more affected by variations in the testing specificity than by variations in testing sensitivity.

### (b) Temporal variations and test heterogeneity

Up to now, we have discussed single viral-load and antibody tests (with and without replacement) but have not considered temporal variations in the number of tests Q, the number of returned positives ${\tilde{Q}}^{+}$, and heterogeneity in FNR and FPR that are associated with different classes (types, manufacturing batches, etc.) of assays. To make our model applicable to empirical time-varying testing data, we use $S_{t}$, $I_{t}$, $R_{t}$ to denote the number of susceptible, infected and removed individuals at time t (or in successive time windows labelled by t), respectively. If K>1 test classes are present, we also include an additional index c∈{1,…,K} in all relevant model parameters. The testing bias and the total number of tests may be both test-class and time-dependent. That is, $b=b_{t,c}$ and $Q=Q_{t,c}$. Test specificity and sensitivity mainly depend on the assay type and not on time. We thus set $\mathrm{FPR}={\mathrm{FPR}}_{c}$ and $\mathrm{FNR}={\mathrm{FNR}}_{c}$.

## 4. Inference of prevalence and application to COVID-19 data

One often wishes to infer the evolution of $I_{t}+R_{t}$ and $S_{t}$, or $I_{t}$ and $S_{t}+R_{t}$ over a given time period from values of $b_{t,c}$, $Q_{t,c}$ and $q_{t,c}$. Since $b_{t,c}$ is difficult to independently ascertain, one may only be able to infer ${\left(f_{\text{b}}\right)}_{t,c}=f(S_{t},I_{t},R_{t},b_{t,c})$. For a single test result ${\tilde{q}}_{t,c}$ (or ${\left({\tilde{f}}_{\text{b}}\right)}_{t,c}$), we can generate the maximum likelihood estimate (MLE) of the bias-modified prevalence ${\left({\hat{f}}_{\text{b}}\right)}_{t,c}$ by setting the measured value ${\left({\tilde{f}}_{\text{b}}\right)}_{t,c}={\bar{\mu }}_{\text{T}}[{\left({\hat{f}}_{\text{b}}\right)}_{t,c}]$ to find

4.1 $${\left({\tilde{f}}_{\text{b}}\right)}_{t,c}={\left({\hat{f}}_{\text{b}}\right)}_{t,c}(1-{\text{FNR}}_{c})+(1-{\left({\hat{f}}_{\text{b}}\right)}_{t,c}){\text{FPR}}_{c}$$

and

4.2 $${\left({\hat{\bar{\sigma }}}_{\text{T}}\right)}_{t,c}^{2}\approx {\left({\bar{\sigma }}_{\text{T}}\right)}_{t,c}^{2}[{\left({\hat{f}}_{\text{b}}\right)}_{t,c}].$$

According to equation (3.23), the variance estimate ${\left({\hat{\bar{\sigma }}}_{\text{T}}\right)}_{t,c}^{2}$ is inversely proportional to the total number of tests Q. Since all other terms in ${\left({\hat{\bar{\sigma }}}_{\text{T}}\right)}_{t,c}^{2}$ are products of quantities with values between 0 and 1, the variance ${\left({\hat{\bar{\sigma }}}_{\text{T}}\right)}_{t,c}^{2}$ approaches zero as Q→∞. Since ${\left({\hat{f}}_{\text{b}}\right)}_{t,c}={\hat{f}}_{t,c}B({\hat{f}}_{t,c},b_{t,c})$, equation (4.1) can be solved for ${\hat{f}}_{t,c}$

4.3 $${\hat{f}}_{t,c}=\frac{{\left({\tilde{f}}_{\text{b}}\right)}_{t,c}-{\text{FPR}}_{c}}{{\text{e}}^{b_{t,c}}[1-{\text{FNR}}_{c}-{\left({\tilde{f}}_{\text{b}}\right)}_{t,c}]+{\left({\tilde{f}}_{\text{b}}\right)}_{t,c}-{\text{FPR}}_{c}}.$$

The posterior distribution $P_{\text{post}}$ over values of f can be found through Bayes’ theorem

4.4 $$P_{\text{post}}(f|{\tilde{Q}}^{+},Q,\text{FPR},\text{FNR})=\frac{P_{\text{TOT}}({\tilde{Q}}^{+}|Q,f,\text{FPR},\text{FNR})P_{0}(f)}{\int_{0}^{1}P_{\text{TOT}}({\tilde{Q}}^{+}|Q,f^{\prime },\text{FPR},\text{FNR})P_{0}(f^{\prime })\;\text{d}f^{\prime }},$$

where $P_{0}(f)$ is a prior distribution over the underlying infection fraction f in the population. For notational brevity, we did not include the indices c and t in equation (4.4). We can again simplify the analysis by using the Gaussian approximation and a simple initial uniform prior, $P_{0}(f<f_{\text{max}}\leq 1)=1/f_{\max}$.

**Figure 3.**
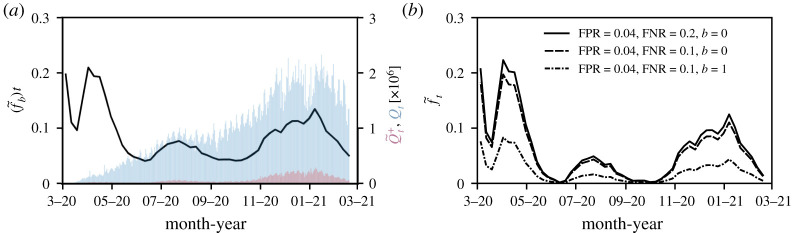
Observed and corrected proportions of positive tests in the USA. (*a*) The solid black line represents the 7-day average of the proportion of positive tests ${\left({\tilde{f}}_{\text{b}}\right)}_{t}={\tilde{Q}}_{t}^{+}/Q_{t}$ in the United States. Blue and red bars show the corresponding total number of daily tests Q and apparent positive tests ${\tilde{Q}}_{t}^{+}$, respectively. (*b*) The corrected proportion of positive tests ${\hat{f}}_{t}$, found by inverting equation (4.1), for different FPR, FNR and bias combinations. (Online version in colour.)

As an example, we collected US testing data [25] from March 2020 to March 2021. Figure 3*a* shows the daily number of observed positive tests ${\tilde{Q}}_{t}^{+}$ (red bars) and the corresponding total daily number of tests $Q_{t}$ (blue bars). The 7-day average of the observed positive testing rate ${\left({\tilde{f}}_{\text{b}}\right)}_{t}={\tilde{Q}}_{t}^{+}/Q_{t}$ is indicated by the black solid line. The first drop in ${\left({\tilde{f}}_{\text{b}}\right)}_{t}$ in March 2020 was associated with the initially very limited number of available SARS-CoV-2 testing infrastructure followed by the ramping up of testing capacity. After new cases surged by the end of March and in April 2020, different types of stay-at-home orders and distancing policies with different durations were implemented across the USA [26]. In June and July 2020, reopening plans were halted and reversed by various jurisdictions to limit the resurgence of COVID-19 [27].

In figure 3*b*, we show the corrected proportion of positive tests ${\hat{f}}_{t}$, found by numerically inverting equation (4.1) for different FPR, FNR and bias combinations. We observe that a small FPR=0.04 shifts values ${\left({\tilde{f}}_{\text{b}}\right)}_{t}\approx 0.05$ towards zero such that the corrected positive testing rate ${\hat{f}}_{t}\approx 0$. Reducing the FNR from 0.2 to 0.1 has only little effect on the corrected proportion of positive tests ${\hat{f}}_{t}$ (solid black and dashed lines in figure 3*b*). Accounting for a positive testing bias of b=1 (i.e. preferential testing of infected and symptomatic individuals by a factor of e), however, markedly changes the inferred ${\hat{f}}_{t}$ (dashed-dotted black line in figure 3*b*). Since the 7-day average of the number of tests Q is about $10^{6}$ in the USA (figure 3*a*), the variance terms ${\left({\hat{\bar{\sigma }}}_{\text{T}}\right)}_{t}^{2}$ are very small compared to the values of ${\hat{f}}_{t}$.

## 5. Inference of bias *b*

One way to estimate the testing bias b is to identify a smaller subset of control tests within a jurisdiction that is believed to be unbiased and compare it with the reported fraction of positive tests obtained via standard (potentially biased) testing procedures. Given this scenario, we can derive a rather complete methodology to estimate bias by formally comparing the statistics of two sets of tests applied to the same population. The first set of control tests with testing parameters $\theta_{0}=\{Q_{0},{\text{FPR}}_{0},{\text{FNR}}_{0}\}$ is known to be unbiased (has prior distribution δ(b)), while the second set is taken with known parameters θ={Q,FPR,FNR}, but unknown testing bias b. For example, the control set may consist of a smaller number $Q_{0}$ of tests that are administered completely randomly, while the second set may be the scaled-up set of tests with $Q>Q_{0}$. Since both sets of tests are applied roughly at the same time to the same overall population, the underlying positive fraction f is assumed to be the same in both test sets. We can then use Bayes’ rule on the first unbiased test set to infer f

5.1 $$P_{\text{post}}(f|{\tilde{f}}_{0},\theta_{0},b=0)=\frac{P_{\text{TOT}}({\tilde{f}}_{0}|f,\theta_{0},b=0)P_{0}(f)}{\int_{0}^{1}P_{\text{TOT}}({\tilde{f}}_{0}|f^{\prime },\theta_{0},b=0)P_{0}(f^{\prime })\;\text{d}f^{\prime }}.$$

The probability distribution over b for a specified value of f can also be constructed from Bayes’ rule

5.2 $$P_{\text{post}}(b|{\tilde{f}}_{\text{b}},f,\theta )=\frac{P_{\text{TOT}}({\tilde{f}}_{\text{b}}|f,\theta ,b)P_{0}(b)}{\int_{-\infty }^{\infty }P_{\text{TOT}}({\tilde{f}}_{\text{b}}|f,\theta ,b^{\prime })P_{0}(b^{\prime })\;\text{d}b^{\prime }},$$

where $P_{0}(\cdot )$ are prior distributions over the relevant parameters. The final distribution over the bias factor, given the two measurements ${\tilde{f}}_{0}$ and ${\tilde{f}}_{\text{b}}$ derived from the two sets of tests with testing parameters $\theta_{0}$ and θ, can be found using

5.3 $$P_{\text{b}}(b|{\tilde{f}}_{\text{b}},{\tilde{f}}_{0},\theta ,\theta_{0})=\int_{0}^{1}P_{\text{post}}(b|{\tilde{f}}_{\text{b}},f,\theta )P_{\text{post}}(f|{\tilde{f}}_{0},\theta_{0},b=0)\text{d}f.$$

Of course, a simpler MLE can also be applied to data by first inferring the most likely value of f from the control test set. We can use the number of positive tests in the control sample $Q_{0}^{+}$ to define the variable ${\tilde{f}}_{0}=Q_{0}^{+}/Q_{0}$. One can then maximize $P_{\text{TOT}}({\tilde{f}}_{0}|f,\theta_{0},b=0)$ with respect to f and use this value $\hat{f}$ in $P_{\text{TOT}}({\tilde{f}}_{\text{b}}|\hat{f},\theta ,b)$. Maximizing $P_{\text{TOT}}({\tilde{f}}_{\text{b}}|\hat{f},\theta ,b)$ with respect to b then gives the MLE estimate $\hat{b}$. We can use random and unbiased sampling results obtained in the German jurisdiction of Gangelt, North Rhine–Westphalia [18]. A total of 600 adult persons with different last names were randomly selected from a population of 12 597 and asked to participate in the study together with their household members. The resulting study comprised of $Q_{0}=919$ subjects who underwent serological and PCR testing between 31 March and 6 April 2020. The specificity and sensitivity corrected, unbiased positive test fraction was determined to be f=15.53% (95% CI 12.31–18.96%). Thus, we use this value as an estimate for the true underlying positivity rate $\hat{f}$. The larger sample taken across North Rhine–Westphalia between 30 March 30 and 5 April 2020 was measured (Q≈25 000) to be ${\tilde{f}}_{\text{b}}\approx 0.1$ [28]. Assuming that this value is also error-corrected, an estimate of the bias $\hat{b}$ in this main testing set can be found by solving ${\tilde{f}}_{\text{b}}\approx 0.1={\bar{\mu }}_{\text{T}}(\hat{f}=0.1553,\hat{b}$, $\text{FPR}=\text{FNR}=0)=\bar{\mu }(\hat{f}=0.1553,\hat{b})$ for $\hat{b}$. We find that the difference between the unbiased positive testing rate of 15.53% and 10% corresponds to a bias of $\hat{b}=-0.50$. This negative bias likely arises because Gangelt was an infection hotspot within the entire North Rhine–Westphalia region, so the control sample was probably not unbiased. For comparison, a higher biased positive testing rate of 20% would lead to an estimated testing bias $\hat{b}=0.31$.

The number of total deaths on 6 April 2020 amounted to 7. Hence, the corresponding estimate of the IFR, the number of disease-induced deaths $D_{t}$ divided by the total number of cases $N_{t}$ at time t, in this jurisdiction on 6 April 2020 was 7/(0.1553×12,597)=0.36% (95% CI 0.29–0.45%) [18]. If only a biased estimate of the proportion of positive cases is known and not the true value f, we can use our framework to distinguish between the true ${\text{IFR}}_{t}=D_{t}/(N_{t}-S_{t})=D_{t}/(fN_{t})$ and the observed infection fatality ratio

5.4 $${\tilde{\text{IFR}}}_{t}=\frac{D_{t}}{\bar{\mu }(f,b)N_{t}}=\frac{D_{t}}{fB(f,b)N_{t}}.$$

**Figure 4.**
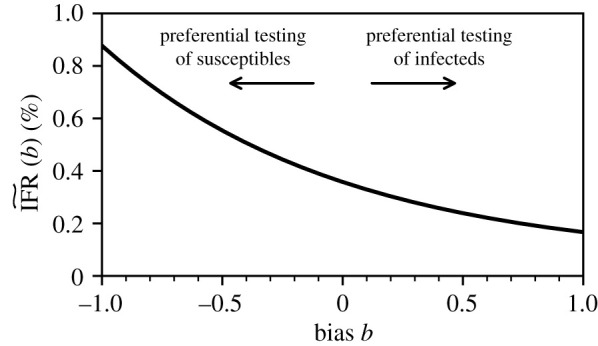
Dependence of the observed IFR on testing bias. The observed infection fatality ratio $\tilde{\mathrm{IFR}}$ (equation (5.4)) as a function of testing bias b. We used the example of the German jurisdiction Gangelt and set $\hat{f}=0.1553$, D=7 and N=12,597. A value b>0 indicates a testing bias towards currently and/or previously infected individuals while susceptible and/or non-infectious individuals are preferentially tested for b<0. Unbiased testing corresponds to b=0.

Figure 4 shows the observed $\tilde{\text{IFR}}$ as a function of testing bias b for the aforementioned example of the German jurisdiction of Gangelt. Values of b>0 correspond to preferential testing of infected individuals and thus lead to an apparently lower $\tilde{\text{IFR}}$. The opposite holds for b<0.

## 6. Summary and conclusion

Radiological testing methods such as chest computed tomography are used sporadically to identify COVID-19-induced pneumonia in patients with negative tests [29]. However, the overwhelming majority of COVID-19 tests are based on serological (or antibody) tests and rapid antigen tests, ELISA and RT-PCR assay [1]. These tests are designed to subsequently output a binary signal, either infected or not. The population statistics of this output are affected by testing errors and bias. False-positive and false-negative rates of serological tests are generally smaller than those of rapid antigen tests and RT-PCR tests. However, serological tests are unable to identify early-stage infections since they are measuring antibody titres that usually develop a few days up to a few weeks after infection. In addition to the occurrence of false positives and false negatives (i.e. type I and type II errors), certain demographic groups (e.g. elderly people or those with comorbidities such as heart and lung diseases) may be overrepresented in testing statistics.

To quantify the impact of both type I/II errors and testing bias on reported COVID-19 case and death numbers, we developed a mathematical framework that describes erroneous and biased sampling (both with and without replacement) from a population of susceptible, infected and removed (i.e. recovered or deceased) individuals. We identify a positive testing bias b>0 with an overrepresentation of previously or currently infected individuals in the study population. Conversely, a negative testing bias b<0 corresponds to an overrepresentation of susceptible and/or non-infectious individuals in the study population. We derived MLEs of the testing-error and testing-bias-corrected fraction of positive tests. Our methods can be also applied to infer the full distribution of corrected positive testing rates over time and for different types of tests across different jurisdictions.

The mathematical quantity that underlies most of our analysis is the proportion of apparent positive tests. As pointed out in [30], the *absolute* number of positive tests may not capture the actual growth of an epidemic due to limitations in testing capacity. Still, many jurisdictions report absolute case numbers without specifying the total number of tests or additional information about test type, date of test and duplicate tests [31], rendering interpretation and application to epidemic surveillance challenging. For a reliable picture of COVID-19 case numbers, more complete testing data, including total number of tests, number of positive tests, test type and date of test, has to be reported and made publicly available at online data repositories. To correct for false positive, false negatives and testing bias in testing statistics (figure 3), it will be also important to further improve estimates of FPR, FNR, and b through in field studies. In particular, estimating the testing bias b requires random sampling studies similar to that carried out in [18]. Finally, while we have presented our analysis in the context of the COVID-19 pandemic, the general results presented in this paper apply to testing and estimation of severity of any infectious disease afflicting a population.

## Data accessibility

Our source codes are publicly available at https://github.com/lubo93/disease-testing.
